# Symptoms and diagnostic delays in bladder cancer with high risk of recurrence: results from a prospective FinnBladder 9 trial

**DOI:** 10.1007/s00345-019-02841-4

**Published:** 2019-06-08

**Authors:** Ville Sell, Otto Ettala, Ileana Montoya Perez, Riikka Järvinen, Tarmo Pekkarinen, Markku Vaarala, Marjo Seppänen, Tapani Liukkonen, Timo Marttila, Sirpa Aaltomaa, Eero Kaasinen, Peter J. Boström

**Affiliations:** 1 Department of Urology, Turku University Hospital, Turku, Finland; 2 Department of Future Technology, University of Turku, Turku, Finland; 3 Department of Urology, Helsinki University Hospital, Helsinki, Finland; 4 Department of Urology, Tampere University Hospital, Tampere, Finland; 5 Department of Urology, Oulu University Hospital, Oulu, Finland; 6 Department of Surgery, Satakunta Central Hospital, Pori, Finland; 7 Department of Surgery, Mikkeli Central Hospital, Mikkeli, Finland; 8 Department of Surgery, Seinäjoki Central Hospital, Seinäjoki, Finland; 9 Department of Urology, Kuopio University Hospital, Kuopio, Finland; 10 Department of Surgery, Hospital of Hyvinkää, Hyvinkää, Finland

**Keywords:** Diagnosis, Symptoms, Diagnostic delay, Hematuria, Bladder cancer

## Abstract

**Purpose:**

To investigate the symptoms and delays in the clinical pathway of bladder cancer (BC).

**Methods:**

This is a substudy of a prospective, randomized, multicenter phase III study (FinnBladder 9, NCT01675219) where the efficacy of photodynamic diagnosis and 6 weekly optimized mitomycin C instillations are studied in pTa bladder cancer with high risk for recurrence. The data of presenting symptoms and critical time points were prospectively collected, and the effect of factors on delays was analyzed.

**Results:**

At the time of analysis, 245 patients were randomized. Analysis included 131 patients with primary bladder cancer and their complete data. Sixty-nine percent had smoking history and 67% presented with macroscopic hematuria. Median patient delay (from symptoms to health-care contact) was 7 days. The median general practice delay (from health-care contact to urology referral) was 8 days. Median time from urology referral to cystoscopy was 23 days and from cystoscopy to TUR-BT 21 days. Total time used in the clinical pathway (from symptom to TUR-BT) was 78 days. Current and former smokers had non-significantly shorter patient-related and general practice delays compared to never smokers. TUR-BT delay was significantly shorter in patients with malignant cytology (16 days) compared to patients with benign cytology (21 days, *p* = 0.03).

**Conclusions:**

Patient-derived delay was short and most of the delay occurred in the referral centers. The majority had macroscopic hematuria as the initial symptom. Surprisingly, current and past smokers were more prone to contact the health-care system compared to never smokers.

## Introduction

Bladder cancer is the second most common urological malignancy and the fourth most common malignancy in males (15th in females) in Finland with approximately 1200 new diagnoses and 300 deaths from the disease in 2015 [1]. Important risk factors for bladder cancer are age, male gender, smoking and occupational exposures with cyclic organic chemicals [2].

Macroscopic hematuria is a common symptom of bladder cancer and is typically present in 80% of patients [3]. Other commonly reported symptoms are urinary frequency, urgency or lower urinary tract symptoms. Despite the fact that hematuria occurs in both genders, women are less likely to be referred to urology [4, 5]. According to some studies, smokers, especially those with microscopic hematuria, undergo less profound assessment [4]. As prolonged hematuria assessment, are associated with worse prognosis; a prompt diagnostic pathway is important to exclude bladder cancer [6, 7].

Recently, the literature of diagnostic delays and hematuria assessment has been systematically reviewed [8]. According to earlier prospective studies, patient-related delays are rather short, often from 1 to 2 weeks and hospital delay is the most significant cause of the total delay [9, 10]. The significance of factors related to diagnostic delays in the clinical pathway of bladder cancer treatment is in some respects controversial and scarce. The systematic review recommended further investigations assessing the effect of age on delays [8]. Controversially, only Mansson et al. have found that elderly patients had longer delays from initial medical consultation to bladder cancer diagnosis [11], but several other studies have reported no consequent differences with varying age [9, 12, 13]. Furthermore, Richards et al. observed that female gender was associated with sub-optimal hematuria assessment. According to large volume studies, female gender is related with delayed hematuria assessment and seems to be more likely managed for urinary tract infection (UTI) during their evaluation [14].

Although smoking is a strong risk factor for bladder cancer, smokers paradoxically appeared less likely to undergo comprehensive assessment [8]. Buteau et al. found no differences in urology referral rates between smokers and never smokers [4], but Elias et al. noticed that smoking was associated with an increased likelihood of having no cystoscopy [15]. However, most studies focus on delays after the patient’s first contact with the health-care system and only a few have assessed all of these factors related to delays.

The aim of the current study is to investigate the initial symptoms and diagnostic delays in the diagnostic pathway of bladder cancer patients in the setting of a prospective randomized multicenter trial.

## Materials and methods

This is a substudy of an ongoing prospective, randomized, multicenter phase III study (FinnBladder 9, ClinicalTrials.gov identifier: NCT01675219). In the study, the efficacy of photodynamic diagnosis TUR-BT (vs. white light TUR-BT) as well as the efficacy of 6 weekly optimized mitomycin C instillations (vs. surveillance) is studied in patents with histologically proven pTa low-grade bladder cancer with high risk for recurrence (multifocal (no. of tumors ≥ 2) or large (≥ 3 cm) primary or recurrent tumors). The “symptoms and diagnostic delays” was a preplanned substudy presented in the original study protocol. Within this preplanning, the specific questionnaires regarding symptoms and delays were designed and prospectively collected.

Eligible patients with signed informed consent were randomized after diagnostic cystoscopy at the outpatient clinic in each of ten attending institutions. Eligibility was re-evaluated after TUR-BT, and if high-grade or invasive (≥ pT1) carcinoma or no BC was observed, the patient was excluded from the main study. In the current substudy, patients were included irrespective of the pathological stage. However, those with recurrent tumors were excluded because of unreliable data concerning symptom initiation.

After randomization, participants filled a comprehensive questionnaire concerning patient characteristics including date of birth, gender, smoking status, the type of presenting symptoms, and specific dates describing the diagnostic pathway. Symptoms were categorized as macroscopic hematuria or other, which included irritation symptoms, bladder outlet obstruction microscopic hematuria, and detection because of other investigations. We reported only macroscopic hematuria, as the initial symptom had to be experienced by the patient and therefore the symptom triggered the contact to the health-care system. Most patients had microscopic hematuria detected when urinary symptoms were investigated, but in three patients asymptomatic microscopic hematuria in routine investigations was the sign triggering further investigations. According to patient-reported time points, two different pre-consultation delays were defined—*patient delay*: time from initiation of symptoms to the first contact with the health-care system; *general practice delay*: time from the first contact with the health-care system to referral to urologist consultation and cystoscopy. Additionally, two diagnostic delays were retrieved from patient charts—*cystoscopy delay*: time between urology referral to cystoscopy; *TUR*-*BT delay*: time between the first cystoscopy and TUR-BT.

Patient characteristics were presented using median and interquartile range (IQR) or number [*n* (%)]. Factors affecting time delays were studied by comparing the medians and the statistical significance was analyzed by Mann–Whitney *U-* and Kruskal–Wallis tests.

## Results

**Fig. 1:**
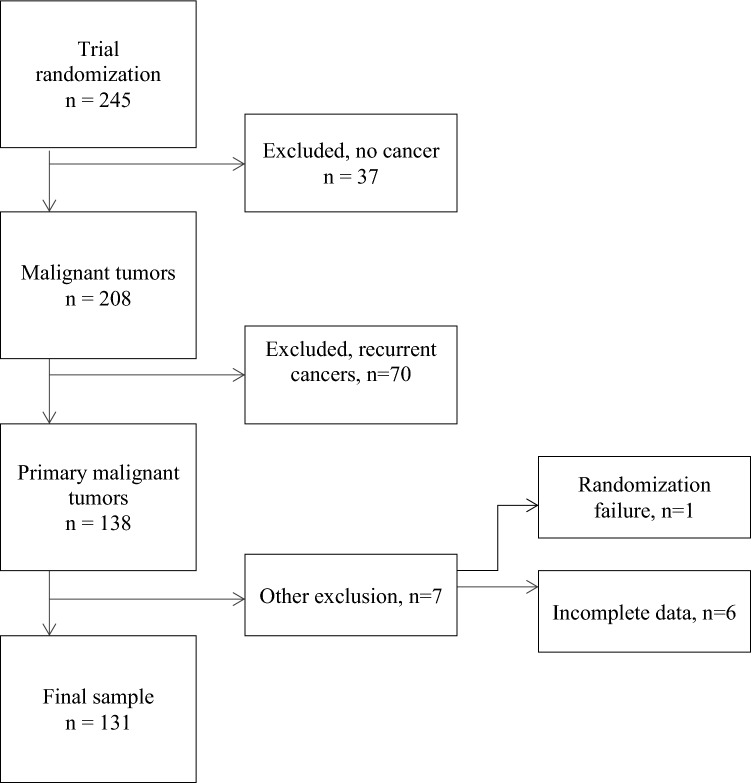
Flowchart of the study population inclusion process

At the time of data analysis for the current substudy, 245 patients were randomized to the study. Accrual and exclusion of the study subjects are presented in Fig. 1. Of the randomized patients, 37 had no cancer, 70 had recurrent tumor, 6 did not complete the questionnaires properly and 1 was regarded as a randomization failure. Therefore, 131 patients with primary bladder cancer and with all questionnaire and clinical data available were included in the analysis.

The baseline patient and tumor characteristics are shown in Table 1. The majority (84%) were men and the mean age was 71 years. Of all patients, 69% had smoking history and most (67%) presented with macroscopic hematuria. Non-muscle invasive tumors were present in 90% of patients. Excluded patients did not have significantly different characteristics when compared to included ones.

**Table 1:** Baseline clinicopathological characteristics

Gender	Male	*N* (%)	110 (84)
Age	Years	Mean (range)	71 (47–94)
Smoking status	Current smoker Former smoker Never smoker Unknown	*N* (%)	26 (20) 64 (49) 22 (17) 19 (14)
Symptoms	Hematuria Other Not reported	*N* (%)	88 (67) 26 (20) 17 (13)
Hospital districts	Helsinki University Hospital Turku University Hospital Tampere University Hospital Oulu University Hospital Kuopio University Hospital	*N* (%)	28 (21) 19 (15) 37 (28) 19 (15) 28 (21)
T-category	pTa pT1 pT2	*N* (%)	79 (60) 38 (29) 14 (11)
Concomitant in situ	Yes No	*N* (%)	10 (8) 121 (92)
Urinary cytology	1–2 3 4–5	*N* (%)	59 (45) 39 (30) 33 (25)

Most patients were referred to urology from public health care (*n* = 71, 54%). Referrals from private physician were non-urologic (*n* = 24, 18%) or urology (*n* = 3, 2%) referrals. Non-urologic referrals from private physicians were mainly from occupational health services. Referrals from other sources were also reported (*n* = 15, 12%).

Median patient delay was 7 days. The median general practice delay was 8 days. Median time from urology referral to cystoscopy was 23 days and the TUR-BT delay was 21 days. Total delay of this clinical pathway from symptom to TUR-BT was 78 days.

Pre-consultation delays, i.e., patient and general practice delays, in different patient sub-groups are presented in Table 2 in days with median (IQR). In patient delay, no differences were found between age and gender. Current and former smokers had non-significantly shorter patient delay compared to never smokers.

**Table 2:** Pre-consultation delays

Characteristics	Descriptions	Patient delay, days Median (IQR)		General practice delay, days Median (IQR)	
Entire cohort	All patients	7 (27)		8 (37)	
Age	< 70 > 70	11 (28) 6 (29)	*p* = 0.80	7 (42) 10 (37)	*p* = 0.99
Gender	Male Female	7 (31) 8 (26)	*p* = 0.99	8 (38) 9 (62)	*p* = 0.88
Smoking status	Smoker^a^ Never	6 (26) 19 (44)	*p* = 0.46	8 (38) 19 (93)	*p* = 0.36
Primary symptom	Hematuria Other	9 (29) 6 (18)	*p* = 0.57	8 (37) 19 (39)	*p* = 1.00
Primary contact	Private health care Public health care	5 (37) 7 (28)	*p* = 0.68	17 (34) 7 (44)	*p* = 0.54

*Patient delay* time from initiation of symptoms to the first contact with the health-care system; *general practice delay* time from the first contact with health-care system to referral to urologist consultation and cystoscopy

^a^Current and former smokers

Similarly, general practice delay showed no differences between age and gender and also current and former smokers had also non-significantly shorter general practice delay. General practice delay was non-significantly shorter in patients presenting with hematuria (8 days) when compared to patients with other symptoms (19 days). Patients referred from public health care had shorter general practice delay (7 days) compared to patients referred from private health care (17 days), although insignificantly. Diagnostic delays, i.e., cystoscopy and TUR-BT delay, in different patient sub-groups are depicted in Table 3.

**Table 3:** Diagnostic delays

Characteristics	Descriptions	Cystoscopy delay, days Median (IQR)		TUR-BT delay, days Median (IQR)	
Entire cohort	All patients	23 (15)		21 (15)	
Age	< 70 > 70	22 (13) 24 (17)	*p* = 0.14	20 (14) 22 (14)	*p* = 0.19
Gender	Male Female	23 (17) 24 (12)	*p* = 0.70	22 (15) 21 (14)	*p* = 0.80
Hospital districts	HYKS TYKS TAYS OYS KYS	21 (19) 26 (17) 25 (16) 17 (12) 23 (9)	*p* = 0.10	26 (11) 19 (19) 21 (15) 20 (22) 22 (21)	*p* = 0.03
Smoking status	Smoker^a^ Never	24 (16) 21 (10)	*p* = 0.20	21 (15) 23 (18)	*p* = 0.37
Urine cytology	1–2 3 4–5	23 (18) 23 (14) 21 (10)	*p* = 0.64	21 (15) 24 (10) 16 (17)	*p* = 0.03

*TUR*-*BT* transurethral resection of bladder tumor, *Cystoscopy delay* time between GP referral to cystoscopy, *TUR*-*BT delay* time between the first cystoscopy and TUR-BT, *HYKS* Helsinki University Hospital district, *TYKS* Turku University Hospital district, *TAYS* Tampere University Hospital district, *OYS* Oulu University Hospital district, *KYS* Kuopio University Hospital district

^a^Current and former smokers

TUR-BT delay showed no differences between age and gender. Current and former smokers had similar TUR-BT delay compared to never smokers. TUR-BT delay was significantly shorter in patients with malignant cytology (16 days) compared to patients with benign cytology (21 days, *p* = 0.03).

## Discussion

In this prospective study, we demonstrate that patient-derived delay was rather short (7 days) and most of the delay occurred in the referral centers. Median time for the entire diagnostic pathway was 78 days. Results in our study are comparable with a Swedish population study [16]. Most patients presented with macroscopic hematuria. Current and past smokers were more prone to contact the health-care system compared to never smokers. Otherwise, no considerable differences were noted in patient delays between different patient cohorts.

The most common presenting symptom of bladder cancer is visible painless hematuria (up to 80% of all BC patients) [3]. Other symptoms related to bladder cancer are unexplained urinary frequency, urgency or irritative voiding symptoms of the lower urinary tract. In our cohort, visible hematuria was the primary symptom in 67% of patients, which is lower than that described in other studies [3]. According to literature, most recent studies concerning symptoms in bladder cancer diagnostics have focused on hematuria, and only few studies have evaluated symptoms more widely. Patients with macroscopic hematuria promptly seek medical advice and also in medical care hematuria is recognized as an alarming symptom meaning shorter referral wait times [9, 10]. Similarly to our study, the recent systematic review assessed that age has no impact on hematuria assessment [8]. It seems that the influence of gender in hematuria patients is complex and widely studied. Female hematuria patients are less likely to be referred to urologic evaluation [4, 5] and they have more pre-referral consultations [17]. Females are also more likely to be managed for UTI during their hematuria evaluation [13, 14]. However, a recent study showed that gender is not a significant independent predictor of delayed hematuria assessment [18], which was in agreement with our investigation as we found no correlation between gender and assessment of hematuria.

Patient delay is unique as it is not directly affected by medical system or health-care professionals. Most evident is that patient delay is rather short and in our investigations the median was 7 days, which is in good agreement with previous studies [9–11]. However, only a few prospective studies have assessed the effect of different factors on prolonged patient delay, and were unable to expose any clinically relevant factors involved. In a retrospective study, Månsson et al. found that hematuria patients had the shortest patient delay [11]. In a large prospective study, Wallace et al. have widely assessed most of the relevant factors and found correlation only in prolonged delays with higher tumor stage and patients with unknown hematuria status [9]. In our study, there were no differences in patient delay regarding hematuria or tumor status; neither did gender disparities occur, which is in agreement with both studies [9, 11]. The impact of patient’s smoking history on patient delay has also been poorly studied. Only Wallace et al. addressed smoking as a risk factor for delayed hematuria assessment and no difference was found between smokers and never smokers [9]. In our study never smokers seemed to be less alarmed than current and former smokers when it comes to contacting health-care providers. Potentially, smokers are more worried about their health, especially when it comes to symptoms suggestive of cancer.

General practice delay is more comprehensively studied and is measured to be rather short [19, 20]. However, the total delay between the onset of hematuria and diagnosis of bladder cancer is long and therefore general practice delay has a key role between the first contact and definitive diagnosis in specialized medical care. Prompt access to urologic investigations and diagnostics is valuable for patients with macroscopic hematuria to decrease diagnostic delays and health-care costs [16]. Hematuria patients are reported to have shorter general practice delays than patients with other symptoms, which was also seen in our study [9, 11]. It is probable that general practitioners are aware of the significance of hematuria as a sign suggestive of bladder cancer and are, therefore, eager to refer the patient to the urologist. Gender disparities did not occur, which amplifies earlier investigations and is in agreement with a recent study [9, 18]. According to our study, current and former smokers are referred to the urologist faster than never smokers, which has not been demonstrated previously. We assume that general practitioners identify smoking as a risk factor for bladder cancer and, therefore, are eager to send the patient to a specialist. Surprisingly enough, patients referred from private outpatient clinics presented with longer pre-consultation delays (i.e., patient and general practice delays). No other previous study has demonstrated such a phenomenon. Health-care system in Finland consists of a large publicly funded health-care system and a much smaller private sector. Treatment of bladder cancer in Finland is mainly performed in public hospitals, but primary diagnosis of bladder cancer can be made both in private and public health care. A general assumption is that this exclusive access to investigations in private health care results in shorter diagnostic delays. One explanation could be the selection bias. Comparing patients referred from public to private health care, those from the private clinics are younger and more frequently never smokers. In addition, although not measured in the current study, the patient–doctor relationship is often longer in the public sector, which may contribute to shorter delays.

Only few studies have described the different aspects of cystoscopy and TUR-BT delays [8]. Most of the studies have a limitation of observing only the total diagnostic delay instead of reporting also these specific sub-delays, i.e., cystoscopy or TUR-BT delay. No differences were found in cystoscopy delay and TUR-BT delay when assessing the influence of age, gender and smoking status. These findings were in coherence with earlier studies, where the total clinical pathway of bladder cancer diagnostic was evaluated [9, 18]. Moreover, in our study, no gender differences were observed when considering the total delay median (men 80 vs. women 76) or only those patients presenting macroscopic hematuria (men, 76 vs. women 76).

In Finland, urine cytology is performed routinely before diagnostic cystoscopy. It is not surprising that positive urine cytology (Papa classes 4–5) was associated with shorter TUR-BT delay, since urine cytology has high sensitivity in high-grade tumors suggestive of urgent treatment. Although this direct finding has not been studied previously, it has been demonstrated that higher tumor stage and larger tumor size are related to shorter delays [9, 11]. There are notable differences in diagnostic delays between the different hospital districts. This is most probably due to the slightly different clinical pathway of bladder cancer diagnostics and treatment.

The major limitations of our study are the small sample size, the recall bias in the pre-consultation delays and the exclusion rate. A significant amount of patients were excluded because of recurring tumors. This was due to the patient’s inability to recall pre-consultation delays before the initial cystoscopy leading to primary diagnosis. However, the recall bias was taken into account early in the study, since participants were asked to complete the questionnaire about the delays just after the initial cystoscopy making the recall bias as minimal as possible. Moreover and most importantly, those excluded patients did not differ significantly from those included. Although the study can be considered as descriptive, the cohort studied can be considered as representative of general bladder cancer patients. The design of the study is a strength. The current trial is a part of a larger, prospective trial. In addition, no other study has evaluated delays in such a manner, especially the patient delay.

## Conclusion

In our study, main delays in diagnosis of bladder cancer take place in specialized medical care. Never smokers and patients presenting with symptoms other than hematuria and patients referred from private outpatient clinics seem to have prolonged pre-consultation delays. However, we found no differences in delays considering age and gender.
